# (*E*)-*N*′-(2,4,5-Trifluorobenzyl­idene)isonicotinohydrazide monohydrate

**DOI:** 10.1107/S1600536810004514

**Published:** 2010-02-10

**Authors:** H. S. Naveenkumar, Amirin Sadikun, Pazilah Ibrahim, Chin Sing Yeap, Hoong-Kun Fun

**Affiliations:** a School of Pharmaceutical Sciences, Universiti Sains Malaysia, 11800 USM, Penang, Malaysia; b X-ray Crystallography Unit, School of Physics, Universiti Sains Malaysia, 11800 USM, Penang, Malaysia

## Abstract

In the Schiff base mol­ecule of the title compound, C_13_H_8_F_3_N_3_O·H_2_O, the benzene ring and the pyridine ring are nearly coplanar, making a dihedral angle of 6.64 (7)°. The mol­ecule exists in an *E* configuration with respect to the C=N double bond. In the crystal structure, mol­ecules are linked *via* the water mol­ecules into two-dimensional planes parallel to the *ab* plane through inter­molecular N—H⋯O, O—H⋯O O—H⋯N and C—H⋯O hydrogen bonds.

## Related literature

For applications of isoniazid (isonicotinylhydrazine) derivatives, see: Janin (2007 ▶); Maccari *et al.* (2005 ▶); Slayden & Barry (2000 ▶); Kahwa *et al.* (1986 ▶). For the preparation of the title compound, see: Lourenco *et al.* (2008 ▶). For related structures, see: Naveenkumar *et al.* (2009 ▶, 2010 ▶); Shi (2005 ▶).
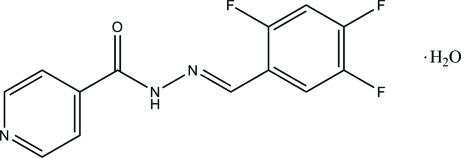


## Experimental

### 

#### Crystal data

C_13_H_8_F_3_N_3_O·H_2_O
*M* *_r_* = 297.24
Triclinic, 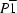

*a* = 4.9241 (1) Å
*b* = 6.3915 (1) Å
*c* = 21.3387 (2) Å
α = 88.616 (1)°
β = 86.556 (1)°
γ = 76.056 (1)°
*V* = 650.58 (2) Å^3^
*Z* = 2
Mo *K*α radiation
μ = 0.13 mm^−1^
*T* = 296 K
0.32 × 0.32 × 0.13 mm

#### Data collection

Bruker SMART APEXII CCD area-detector diffractometer
Absorption correction: multi-scan (*SADABS*; Bruker, 2009 ▶) *T*_min_ = 0.958, *T*_max_ = 0.984
14710 measured reflections
4024 independent reflections
2730 reflections with *I* > 2σ(*I*)
*R*_int_ = 0.022

#### Refinement

*R*[*F* ^2^ > 2σ(*F* ^2^)] = 0.046
*wR*(*F* ^2^) = 0.146
*S* = 1.05
4024 reflections
230 parameters
All H-atom parameters refined
Δρ_max_ = 0.27 e Å^−3^
Δρ_min_ = −0.22 e Å^−3^

### 

Data collection: *APEX2* (Bruker, 2009 ▶); cell refinement: *SAINT* (Bruker, 2009 ▶); data reduction: *SAINT*; program(s) used to solve structure: *SHELXTL* (Sheldrick, 2008 ▶); program(s) used to refine structure: *SHELXTL*; molecular graphics: *SHELXTL*; software used to prepare material for publication: *SHELXTL* and *PLATON* (Spek, 2009 ▶).

## Supplementary Material

Crystal structure: contains datablocks global, I. DOI: 10.1107/S1600536810004514/sj2725sup1.cif

Structure factors: contains datablocks I. DOI: 10.1107/S1600536810004514/sj2725Isup2.hkl

Additional supplementary materials:  crystallographic information; 3D view; checkCIF report

## Figures and Tables

**Table 1:** Hydrogen-bond geometry (Å, °)

*D*—H⋯*A*	*D*—H	H⋯*A*	*D*⋯*A*	*D*—H⋯*A*
N2—H1*N*2⋯O1*W*	0.883 (19)	1.921 (19)	2.7910 (13)	168.1 (16)
O1*W*—H1*W*1⋯O1^i^	0.79 (2)	2.03 (2)	2.8263 (17)	176.3 (18)
O1*W*—H2*W*1⋯O1^ii^	0.84 (2)	2.19 (2)	2.9670 (17)	155 (2)
O1*W*—H2*W*1⋯N1^ii^	0.84 (2)	2.47 (2)	3.1024 (14)	133.7 (19)
C7—H7*A*⋯O1*W*	0.967 (13)	2.492 (14)	3.2576 (16)	135.9 (12)
C13—H13*A*⋯O1*W*	0.963 (19)	2.427 (18)	3.3308 (19)	156.2 (14)

Symmetry codes: (i) 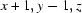; (ii) 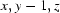.

**Fig. 1.**
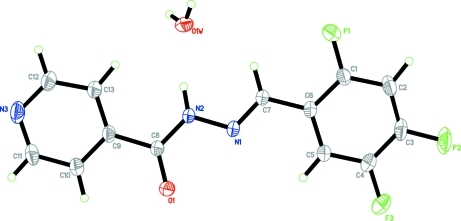
The molecular structure of (I) with atom labels and 50% probability ellipsoids for non-H atoms.

**Fig. 2.**
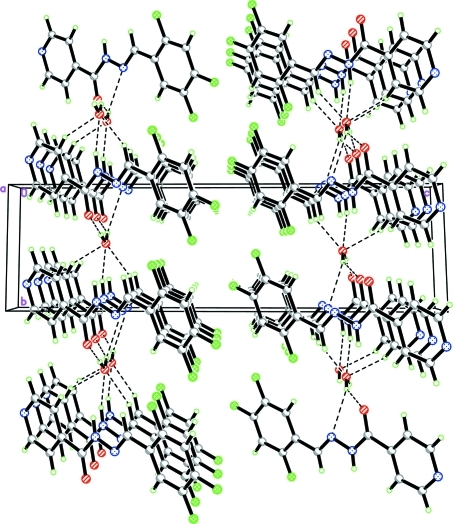
The crystal packing of (I), viewed down the a axis, showing the molecules are linked into 2-dimensional planes parallel to the ab plane. Intermolecular hydrogen bonds are shown as dashed lines.

## supplementary crystallographic information

### Comment

In the search for new biologically active compounds, isoniazid (isonicotinylhydrazine)  derivatives have been found to possess potential tuberculostatic activity (Janin, 2007; Maccari *et al.*, 2005; Slayden & Barry, 2000).  As  part of our current work on the  synthesis of (*E*)-*N*'-substituted isonicotinohydrazide derivatives, in this paper we present the crystal structure of the title compound, (I).

The asymmetric unit consists of one Schiff base molecule and one water molecule (Fig. 1). The geometric parameters are comparable to those related structures (Naveenkumar *et al.*, 2009, 2010; Shi, 2005). The molecule is nearly coplanar with a dihedral angle between the benzene ring and the pyridine ring of 6.64 (7)°. The molecule exists in an *E* configuration with respect to the C7=N1 double bond. In the crystal structure, the water molecules link the molecules into two-dimensional planes parallel to the *ab* plane through intermolecular N–H···O, O–H···O O–H···N and C–H···O hydrogen bonds (Fig. 2, Table 1).

### Experimental

The isoniazid derivative was prepared following a procedure reported by Lourenco *et al.*, (2008).  2,4,5-triflurobenzaldehyde (1.0 eq) was added to isoniazid (1.0 eq) in ethanol/water. After stirring for 1-3 h at room temperature, the resulting mixture was concentrated under reduced pressure. The residue was purified by washing with cold ethanol and diethyl ether to give the pure derivative.  Colourless single crystals suitable for X-ray analysis were obtained by re-crystallization from methanol.

### Refinement

All hydrogen atoms were located from the difference Fourier map and refined freely.

### Crystal data

**Table:**

C_13_H_8_F_3_N_3_O·H_2_O	*Z* = 2
*M**_r_* = 297.24	*F*(000) = 304
Triclinic, *P*1	*D*_x_ = 1.517 Mg m^−^^3^
Hall symbol: -P 1	Mo *K*α radiation, λ = 0.71073 Å
*a* = 4.9241 (1) Å	Cell parameters from 6031 reflections
*b* = 6.3915 (1) Å	θ = 2.9–30.8°
*c* = 21.3387 (2) Å	µ = 0.13 mm^−^^1^
α = 88.616 (1)°	*T* = 296 K
β = 86.556 (1)°	Plate, colourless
γ = 76.056 (1)°	0.32 × 0.32 × 0.13 mm
*V* = 650.58 (2) Å^3^	

### Data collection

**Table:**

Bruker SMART APEXII CCD area-detector diffractometer	4024 independent reflections
Radiation source: fine-focus sealed tube	2730 reflections with *I* > 2σ(*I*)
graphite	*R*_int_ = 0.022
φ and ω scans	θ_max_ = 30.8°, θ_min_ = 2.9°
Absorption correction: multi-scan (*SADABS*; Bruker, 2009)	*h* = −6→7
*T*_min_ = 0.958, *T*_max_ = 0.984	*k* = −9→9
14710 measured reflections	*l* = −30→29

### Refinement

**Table:**

Refinement on *F*^2^	Primary atom site location: structure-invariant direct methods
Least-squares matrix: full	Secondary atom site location: difference Fourier map
*R*[*F*^2^ > 2σ(*F*^2^)] = 0.046	Hydrogen site location: inferred from neighbouring sites
*wR*(*F*^2^) = 0.146	All H-atom parameters refined
*S* = 1.05	*w* = 1/[σ^2^(*F*_o_^2^) + (0.0764*P*)^2^ + 0.0515*P*] where *P* = (*F*_o_^2^ + 2*F*_c_^2^)/3
4024 reflections	(Δ/σ)_max_ < 0.001
230 parameters	Δρ_max_ = 0.27 e Å^−^^3^
0 restraints	Δρ_min_ = −0.22 e Å^−^^3^

### Special details

**Table:**

Geometry. All esds (except the esd in the dihedral angle between two l.s. planes) are estimated using the full covariance matrix. The cell esds are taken into account individually in the estimation of esds in distances, angles and torsion angles; correlations between esds in cell parameters are only used when they are defined by crystal symmetry. An approximate (isotropic) treatment of cell esds is used for estimating esds involving l.s. planes.
Refinement. Refinement of F^2^ against ALL reflections. The weighted R-factor wR and goodness of fit S are based on F^2^, conventional R-factors R are based on F, with F set to zero for negative F^2^. The threshold expression of F^2^ > 2sigma(F^2^) is used only for calculating R-factors(gt) etc. and is not relevant to the choice of reflections for refinement. R-factors based on F^2^ are statistically about twice as large as those based on F, and R- factors based on ALL data will be even larger.

### Fractional atomic coordinates and isotropic or equivalent isotropic displacement parameters (Å^2^)

**Table:**

	*x*	*y*	*z*	*U*_iso_*/*U*_eq_	
F1	1.20930 (18)	0.63383 (14)	0.34682 (4)	0.0667 (3)	
F2	1.2581 (2)	1.19398 (18)	0.48033 (4)	0.0824 (3)	
F3	0.8111 (2)	1.44958 (15)	0.42966 (5)	0.0834 (3)	
O1	0.1646 (2)	1.24750 (13)	0.19113 (5)	0.0552 (3)	
N1	0.5532 (2)	1.00798 (15)	0.25981 (4)	0.0386 (2)	
N2	0.4249 (2)	0.91747 (15)	0.21550 (4)	0.0377 (2)	
N3	−0.1882 (3)	0.7926 (2)	0.04246 (6)	0.0592 (3)	
C1	1.1071 (3)	0.8366 (2)	0.36730 (6)	0.0431 (3)	
C2	1.2389 (3)	0.9080 (3)	0.41488 (6)	0.0532 (3)	
C3	1.1375 (3)	1.1151 (3)	0.43436 (6)	0.0533 (3)	
C4	0.9082 (3)	1.2479 (2)	0.40781 (6)	0.0520 (3)	
C5	0.7792 (3)	1.1753 (2)	0.36057 (6)	0.0465 (3)	
C6	0.8779 (2)	0.96531 (19)	0.33900 (5)	0.0381 (3)	
C7	0.7429 (2)	0.87952 (19)	0.28944 (5)	0.0391 (3)	
C8	0.2279 (2)	1.05161 (17)	0.18276 (5)	0.0381 (3)	
C9	0.0888 (2)	0.95209 (18)	0.13457 (5)	0.0381 (3)	
C10	−0.1271 (3)	1.0858 (2)	0.10359 (6)	0.0469 (3)	
C11	−0.2587 (3)	0.9994 (3)	0.05883 (6)	0.0549 (3)	
C12	0.0208 (4)	0.6670 (3)	0.07203 (7)	0.0645 (4)	
C13	0.1642 (3)	0.7368 (2)	0.11825 (7)	0.0555 (4)	
O1W	0.6042 (3)	0.46898 (15)	0.22162 (5)	0.0567 (3)	
H2A	1.388 (4)	0.807 (3)	0.4315 (9)	0.079 (5)*	
H5A	0.619 (4)	1.275 (3)	0.3436 (8)	0.069 (5)*	
H7A	0.798 (3)	0.727 (2)	0.2805 (7)	0.054 (4)*	
H10A	−0.178 (3)	1.237 (3)	0.1110 (7)	0.061 (4)*	
H11A	−0.398 (4)	1.092 (3)	0.0388 (8)	0.069 (5)*	
H12A	0.087 (4)	0.519 (3)	0.0585 (9)	0.084 (6)*	
H13A	0.316 (4)	0.636 (3)	0.1371 (8)	0.073 (5)*	
H1N2	0.479 (3)	0.776 (3)	0.2116 (8)	0.067 (5)*	
H1W1	0.759 (4)	0.402 (3)	0.2127 (8)	0.071 (6)*	
H2W1	0.499 (4)	0.384 (4)	0.2232 (10)	0.099 (7)*	

### Atomic displacement parameters (Å^2^)

**Table:**

	*U*^11^	*U*^22^	*U*^33^	*U*^12^	*U*^13^	*U*^23^
F1	0.0610 (5)	0.0571 (5)	0.0724 (6)	0.0110 (4)	−0.0253 (4)	−0.0153 (4)
F2	0.0779 (6)	0.1120 (8)	0.0649 (6)	−0.0278 (6)	−0.0330 (5)	−0.0306 (5)
F3	0.0999 (8)	0.0612 (5)	0.0897 (7)	−0.0114 (5)	−0.0295 (6)	−0.0347 (5)
O1	0.0565 (6)	0.0337 (4)	0.0772 (6)	−0.0068 (4)	−0.0346 (5)	−0.0034 (4)
N1	0.0405 (5)	0.0378 (5)	0.0402 (5)	−0.0114 (4)	−0.0145 (4)	−0.0050 (4)
N2	0.0408 (5)	0.0331 (5)	0.0412 (5)	−0.0087 (4)	−0.0177 (4)	−0.0039 (4)
N3	0.0653 (8)	0.0712 (8)	0.0501 (6)	−0.0285 (6)	−0.0241 (5)	−0.0041 (6)
C1	0.0381 (6)	0.0491 (7)	0.0410 (6)	−0.0062 (5)	−0.0103 (5)	−0.0040 (5)
C2	0.0407 (7)	0.0730 (9)	0.0446 (7)	−0.0073 (6)	−0.0172 (5)	−0.0031 (6)
C3	0.0490 (7)	0.0758 (9)	0.0409 (6)	−0.0220 (7)	−0.0140 (5)	−0.0131 (6)
C4	0.0593 (8)	0.0503 (7)	0.0499 (7)	−0.0161 (6)	−0.0125 (6)	−0.0129 (5)
C5	0.0492 (7)	0.0431 (6)	0.0480 (7)	−0.0086 (5)	−0.0183 (5)	−0.0049 (5)
C6	0.0377 (6)	0.0422 (6)	0.0367 (5)	−0.0119 (4)	−0.0110 (4)	−0.0020 (4)
C7	0.0411 (6)	0.0361 (5)	0.0415 (6)	−0.0091 (4)	−0.0139 (5)	−0.0039 (4)
C8	0.0372 (6)	0.0355 (5)	0.0442 (6)	−0.0107 (4)	−0.0138 (5)	−0.0013 (4)
C9	0.0378 (6)	0.0433 (6)	0.0375 (6)	−0.0153 (5)	−0.0126 (4)	−0.0005 (4)
C10	0.0449 (7)	0.0509 (7)	0.0451 (7)	−0.0084 (5)	−0.0161 (5)	−0.0017 (5)
C11	0.0484 (7)	0.0724 (9)	0.0459 (7)	−0.0138 (7)	−0.0216 (6)	0.0011 (6)
C12	0.0858 (11)	0.0528 (8)	0.0630 (9)	−0.0250 (8)	−0.0335 (8)	−0.0060 (7)
C13	0.0671 (9)	0.0427 (7)	0.0603 (8)	−0.0132 (6)	−0.0322 (7)	−0.0036 (6)
O1W	0.0608 (7)	0.0322 (4)	0.0771 (7)	−0.0067 (4)	−0.0194 (5)	−0.0063 (4)

### Geometric parameters (Å, °)

**Table:**

F1—C1	1.3454 (15)	C5—C6	1.3922 (17)
F2—C3	1.3445 (13)	C5—H5A	0.969 (18)
F3—C4	1.3463 (16)	C6—C7	1.4660 (14)
O1—C8	1.2299 (13)	C7—H7A	0.965 (15)
N1—C7	1.2737 (15)	C8—C9	1.5025 (14)
N1—N2	1.3791 (11)	C9—C13	1.3835 (17)
N2—C8	1.3477 (14)	C9—C10	1.3843 (17)
N2—H1N2	0.881 (18)	C10—C11	1.3821 (17)
N3—C12	1.326 (2)	C10—H10A	0.953 (16)
N3—C11	1.3324 (19)	C11—H11A	0.911 (19)
C1—C2	1.3800 (17)	C12—C13	1.3882 (17)
C1—C6	1.3877 (16)	C12—H12A	0.966 (19)
C2—C3	1.363 (2)	C13—H13A	0.963 (18)
C2—H2A	0.93 (2)	O1W—H1W1	0.79 (2)
C3—C4	1.381 (2)	O1W—H2W1	0.83 (2)
C4—C5	1.3699 (16)		
			
C7—N1—N2	116.55 (9)	N1—C7—C6	118.93 (10)
C8—N2—N1	117.36 (9)	N1—C7—H7A	121.8 (9)
C8—N2—H1N2	126.2 (11)	C6—C7—H7A	119.2 (9)
N1—N2—H1N2	116.5 (11)	O1—C8—N2	122.04 (9)
C12—N3—C11	116.24 (11)	O1—C8—C9	120.84 (10)
F1—C1—C2	118.25 (11)	N2—C8—C9	117.12 (9)
F1—C1—C6	118.70 (10)	C13—C9—C10	117.67 (10)
C2—C1—C6	123.04 (12)	C13—C9—C8	124.60 (10)
C3—C2—C1	117.80 (12)	C10—C9—C8	117.72 (10)
C3—C2—H2A	126.1 (11)	C11—C10—C9	119.15 (12)
C1—C2—H2A	116.1 (11)	C11—C10—H10A	119.8 (9)
F2—C3—C2	120.48 (12)	C9—C10—H10A	121.0 (9)
F2—C3—C4	118.53 (13)	N3—C11—C10	123.93 (13)
C2—C3—C4	120.99 (11)	N3—C11—H11A	119.1 (11)
F3—C4—C5	120.41 (12)	C10—C11—H11A	116.9 (11)
F3—C4—C3	118.89 (11)	N3—C12—C13	124.42 (14)
C5—C4—C3	120.70 (12)	N3—C12—H12A	117.9 (11)
C4—C5—C6	120.06 (12)	C13—C12—H12A	117.5 (11)
C4—C5—H5A	117.3 (10)	C9—C13—C12	118.58 (13)
C6—C5—H5A	122.7 (10)	C9—C13—H13A	121.8 (10)
C1—C6—C5	117.41 (10)	C12—C13—H13A	119.6 (10)
C1—C6—C7	120.67 (11)	H1W1—O1W—H2W1	108.0 (19)
C5—C6—C7	121.89 (10)		
			
C7—N1—N2—C8	−178.64 (11)	N2—N1—C7—C6	−177.37 (10)
F1—C1—C2—C3	178.60 (12)	C1—C6—C7—N1	−172.44 (12)
C6—C1—C2—C3	−0.2 (2)	C5—C6—C7—N1	9.12 (19)
C1—C2—C3—F2	−179.70 (12)	N1—N2—C8—O1	0.66 (18)
C1—C2—C3—C4	0.8 (2)	N1—N2—C8—C9	−179.99 (9)
F2—C3—C4—F3	−1.2 (2)	O1—C8—C9—C13	174.03 (13)
C2—C3—C4—F3	178.33 (14)	N2—C8—C9—C13	−5.32 (19)
F2—C3—C4—C5	179.61 (13)	O1—C8—C9—C10	−4.90 (18)
C2—C3—C4—C5	−0.8 (2)	N2—C8—C9—C10	175.74 (11)
F3—C4—C5—C6	−178.86 (13)	C13—C9—C10—C11	0.8 (2)
C3—C4—C5—C6	0.3 (2)	C8—C9—C10—C11	179.78 (12)
F1—C1—C6—C5	−179.11 (12)	C12—N3—C11—C10	0.3 (2)
C2—C1—C6—C5	−0.3 (2)	C9—C10—C11—N3	−1.0 (2)
F1—C1—C6—C7	2.38 (18)	C11—N3—C12—C13	0.7 (3)
C2—C1—C6—C7	−178.84 (12)	C10—C9—C13—C12	0.1 (2)
C4—C5—C6—C1	0.3 (2)	C8—C9—C13—C12	−178.85 (13)
C4—C5—C6—C7	178.75 (12)	N3—C12—C13—C9	−0.9 (3)

### Hydrogen-bond geometry (Å, °)

**Table:**

*D*—H···*A*	*D*—H	H···*A*	*D*···*A*	*D*—H···*A*
N2—H1N2···O1W	0.883 (19)	1.921 (19)	2.7910 (13)	168.1 (16)
O1W—H1W1···O1^i^	0.79 (2)	2.03 (2)	2.8263 (17)	176.3 (18)
O1W—H2W1···O1^ii^	0.84 (2)	2.19 (2)	2.9670 (17)	155 (2)
O1W—H2W1···N1^ii^	0.84 (2)	2.47 (2)	3.1024 (14)	133.7 (19)
C7—H7A···O1W	0.967 (13)	2.492 (14)	3.2576 (16)	135.9 (12)
C13—H13A···O1W	0.963 (19)	2.427 (18)	3.3308 (19)	156.2 (14)

Symmetry codes: (i) *x*+1, *y*−1, *z*; (ii) *x*, *y*−1, *z*.
